# (2*S*,4a*R*,6a*R*,7*R*,9*S*,10a*S*,10b*R*)-7-Carb­oxy-2-(3-fur­yl)-6a,10b-dimethyl-4,10-dioxoperhydro­benzo[*f*]isochromen-9-yl acetate

**DOI:** 10.1107/S1600536809002074

**Published:** 2009-02-06

**Authors:** Paulo Carvalho, Lukasz M. Kutrzeba, Jordan K. Zjawiony, Mitchell A. Avery

**Affiliations:** a Department of Medicinal Chemistry, University of Mississippi, 417 Faser Hall, University, MS 38677, USA; b Department of Pharmacognosy, School of Pharmacy, University of Mississippi, University, MS 38677, USA; c National Center for Natural Products Research, Research Institute of Pharmaceutical Sciences, School of Pharmacy, University of Mississippi, University, MS 38677, USA; d Department of Chemistry and Biochemistry, University of Mississippi, University, MS 38677, USA

## Abstract

The asymmetric unit of the title compound, C_22_H_26_O_8_, contains two crystallographically independent mol­ecules with closely comparable conformations (r.m.s. overlay = 0.54 Å for 30 non-H atoms). All six-membered rings display chair conformations, with a slight distortion for the lactone ring. The mol­ecules are connected by O—H⋯O hydrogen bonds into chains along [010], with the independent mol­ecules segregated into separate chains. The two mol­ecules in the asymmetric unit face each other in a head-to-tail fashion, with the furan ring of one mol­ecule turned towards the carboxylic acid terminal of the other mol­ecule.

## Related literature

For the biosynthesis of Salvinorin A, see: Kutrzeba *et al.* (2007 ▶). For the isolation of Salvinorin A and further synthesis details, see: Lee, Karnati *et al.* (2005 ▶); Lee, He *et al.* (2005 ▶); Stewart (2005 ▶). For details on epimerization at the C-8 stereogenic center, see: Harding *et al.* (2005 ▶).
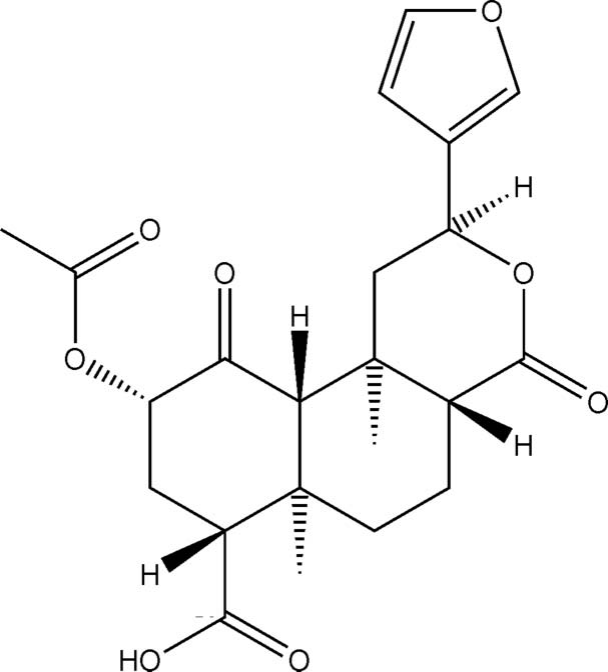


## Experimental

### 

#### Crystal data

C_22_H_26_O_8_
*M* *_r_* = 418.43
Monoclinic, 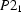

*a* = 11.2735 (6) Å
*b* = 16.8015 (9) Å
*c* = 11.3765 (6) Å
β = 111.934 (3)°
*V* = 1998.86 (18) Å^3^
*Z* = 4
Cu *K*α radiation
μ = 0.89 mm^−1^
*T* = 100 (2) K
0.19 × 0.12 × 0.09 mm

#### Data collection

Bruker APEXII CCD diffractometer
Absorption correction: none
33644 measured reflections
7269 independent reflections
6962 reflections with *I* > 2σ(*I*)
*R*_int_ = 0.034

#### Refinement

*R*[*F* ^2^ > 2σ(*F* ^2^)] = 0.029
*wR*(*F* ^2^) = 0.074
*S* = 1.10
7269 reflections
549 parameters
1 restraint
H-atom parameters constrained
Δρ_max_ = 0.22 e Å^−3^
Δρ_min_ = −0.18 e Å^−3^
Absolute structure: Flack (1983 ▶), 3360 Friedel pairs
Flack parameter: −0.09 (13)

### 

Data collection: *APEX2* (Bruker, 2005 ▶); cell refinement: *SAINT* (Bruker, 2005 ▶); data reduction: *SAINT*; program(s) used to solve structure: *SHELXS97* (Sheldrick, 2008 ▶); program(s) used to refine structure: *SHELXL97* (Sheldrick, 2008 ▶); molecular graphics: *SHELXTL* (Sheldrick, 2008 ▶); software used to prepare material for publication: *SHELXTL* .

## Supplementary Material

Crystal structure: contains datablocks I, global. DOI: 10.1107/S1600536809002074/bi2338sup1.cif

Structure factors: contains datablocks I. DOI: 10.1107/S1600536809002074/bi2338Isup2.hkl

Additional supplementary materials:  crystallographic information; 3D view; checkCIF report

## Figures and Tables

**Table 1:** Hydrogen-bond geometry (Å, °)

*D*—H⋯*A*	*D*—H	H⋯*A*	*D*⋯*A*	*D*—H⋯*A*
O8—H8*A*⋯O6^i^	0.82	1.92	2.7264 (18)	168
O8′—H8′⋯O3′^ii^	0.82	1.92	2.7308 (17)	172

Symmetry code: (i) 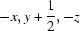; (ii) 
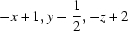
.

**Fig. 1.**
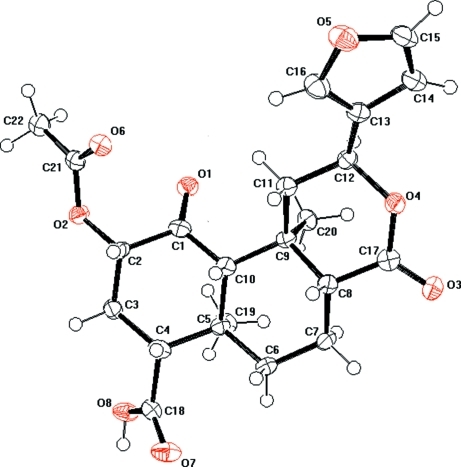
One molecule in the asymmetric unit of the title compound with displacement ellipsoids drawn at the 50% probability level for non-H atoms. The second molecule is closely comparable.

## supplementary crystallographic information

### Comment

Diterpenoid salvinorin A is a potent kappa opioid agonist isolated from Mexican mint *Salvia divinorum*. The biosynthesis of this natural product was studied using synthetic analogues of salvinorin A modified at C-2 and C-18 as chemical probes for *in vitro* enzymatic reactions. Salvinorin A and its C-8 epimeric counterpart revealed low affinity to the kappa-opioid receptor.

### Experimental

The starting material for synthesis, salvinorin A, was isolated from dry plant material, and purified according to a previously published procedure (Stewart, 2005). Salvinorin A acid was synthesized following Lee, He *et al.* (2005), by using LiI in pyridine as a selective hydrolyzing agent of C-4 methyl ester.

### Refinement

All H atoms were visible in difference maps, but were placed geometrically and treated as riding atoms for refinement, with the following constraints: C—H = 0.93 Å, *U*_iso_(H) = 1.2*U*_eq_(C) for C*sp*^2^, C—H = 0.98 Å, *U*_iso_(H) = 1.2*U*_eq_(C) for CH, C—H = 0.97 Å, *U*_iso_(H) = 1.2*U*_eq_(C) for CH_2_, C—H = 0.96 Å, *U*_iso_(H) = 1.5*U*_eq_(C) CH_3_, O—H = 0.82 Å, *U*_iso_(H) = 1.5*U*_eq_(O) for OH.

### Crystal data

**Table:**

C_22_H_26_O_8_	*F*(000) = 888
*M**_r_* = 418.43	*D*_x_ = 1.390 Mg m^−^^3^
Monoclinic, *P*2_1_	Cu *K*α radiation, λ = 1.54178 Å
Hall symbol: P 2yb	Cell parameters from 9847 reflections
*a* = 11.2735 (6) Å	θ = 4.2–69.3°
*b* = 16.8015 (9) Å	µ = 0.89 mm^−^^1^
*c* = 11.3765 (6) Å	*T* = 100 K
β = 111.934 (3)°	Block, colourless
*V* = 1998.86 (18) Å^3^	0.19 × 0.12 × 0.09 mm
*Z* = 4	

### Data collection

**Table:**

Bruker APEXII CCD diffractometer	6962 reflections with *I* > 2σ(*I*)
Radiation source: fine-focus sealed tube, Siemens KFF Cu 2 K90	*R*_int_ = 0.034
graphite	θ_max_ = 69.7°, θ_min_ = 4.2°
φ and ω scans	*h* = −13→13
33644 measured reflections	*k* = −20→20
7269 independent reflections	*l* = −13→13

### Refinement

**Table:**

Refinement on *F*^2^	Secondary atom site location: difference Fourier map
Least-squares matrix: full	Hydrogen site location: inferred from neighbouring sites
*R*[*F*^2^ > 2σ(*F*^2^)] = 0.029	H-atom parameters constrained
*wR*(*F*^2^) = 0.074	*w* = 1/[σ^2^(*F*_o_^2^) + (0.0314*P*)^2^ + 0.6446*P*] where *P* = (*F*_o_^2^ + 2*F*_c_^2^)/3
*S* = 1.10	(Δ/σ)_max_ = 0.001
7269 reflections	Δρ_max_ = 0.22 e Å^−^^3^
549 parameters	Δρ_min_ = −0.17 e Å^−^^3^
1 restraint	Absolute structure: Flack (1983), 3360 Friedel pairs
Primary atom site location: structure-invariant direct methods	Flack parameter: −0.09 (13)

### Special details

**Table:**

Geometry. All e.s.d.'s (except the e.s.d. in the dihedral angle between two l.s. planes) are estimated using the full covariance matrix. The cell e.s.d.'s are taken into account individually in the estimation of e.s.d.'s in distances, angles and torsion angles; correlations between e.s.d.'s in cell parameters are only used when they are defined by crystal symmetry. An approximate (isotropic) treatment of cell e.s.d.'s is used for estimating e.s.d.'s involving l.s. planes.
Refinement. Refinement of *F*^2^ against ALL reflections. The weighted *R*-factor *wR* and goodness of fit *S* are based on *F*^2^, conventional *R*-factors *R* are based on *F*, with *F* set to zero for negative *F*^2^. The threshold expression of *F*^2^ > σ(*F*^2^) is used only for calculating *R*-factors(gt) *etc*. and is not relevant to the choice of reflections for refinement. *R*-factors based on *F*^2^ are statistically about twice as large as those based on *F*, and *R*- factors based on ALL data will be even larger.

### Fractional atomic coordinates and isotropic or equivalent isotropic displacement parameters (Å^2^)

**Table:**

	*x*	*y*	*z*	*U*_iso_*/*U*_eq_	
C17	0.15137 (16)	0.28832 (12)	0.49869 (18)	0.0206 (4)	
C21	−0.01037 (17)	0.36406 (10)	−0.21255 (17)	0.0184 (4)	
C10	0.05570 (16)	0.40092 (10)	0.18239 (17)	0.0171 (4)	
H10	0.1460	0.4079	0.1953	0.021*	
C2	0.02643 (18)	0.44739 (11)	−0.03640 (17)	0.0200 (4)	
H2	0.1187	0.4446	−0.0167	0.024*	
C13	0.22704 (17)	0.12615 (11)	0.32982 (18)	0.0209 (4)	
C3	−0.00836 (19)	0.53085 (11)	−0.00734 (19)	0.0216 (4)	
H3A	−0.1005	0.5356	−0.0341	0.026*	
H3B	0.0208	0.5699	−0.0533	0.026*	
C2'	0.52134 (17)	0.21987 (11)	0.57588 (17)	0.0184 (4)	
H2'	0.4293	0.2214	0.5260	0.022*	
C7	0.07910 (19)	0.43150 (12)	0.44293 (18)	0.0230 (4)	
H7A	−0.0087	0.4227	0.4347	0.028*	
H7B	0.1289	0.4445	0.5307	0.028*	
C1	−0.01037 (16)	0.38607 (10)	0.04262 (18)	0.0184 (4)	
C14	0.25349 (19)	0.05608 (11)	0.4068 (2)	0.0260 (4)	
H14	0.2213	0.0437	0.4688	0.031*	
C6	0.08460 (18)	0.50046 (11)	0.35771 (18)	0.0220 (4)	
H6A	0.1734	0.5116	0.3723	0.026*	
H6B	0.0490	0.5475	0.3816	0.026*	
C20	−0.08067 (17)	0.30143 (11)	0.25474 (18)	0.0200 (4)	
H20A	−0.1174	0.2710	0.1782	0.030*	
H20B	−0.1339	0.3466	0.2514	0.030*	
H20C	−0.0744	0.2688	0.3261	0.030*	
C12	0.13705 (17)	0.19141 (11)	0.33016 (17)	0.0198 (4)	
H12	0.0513	0.1681	0.3033	0.024*	
C5	0.01218 (16)	0.48472 (11)	0.21544 (17)	0.0188 (4)	
C8	0.13200 (17)	0.35598 (11)	0.40574 (17)	0.0194 (4)	
H8	0.2173	0.3698	0.4079	0.023*	
C22	−0.09694 (18)	0.34694 (12)	−0.34524 (18)	0.0229 (4)	
H22A	−0.0585	0.3075	−0.3809	0.034*	
H22B	−0.1110	0.3949	−0.3946	0.034*	
H22C	−0.1772	0.3274	−0.3459	0.034*	
C9	0.05392 (16)	0.33013 (11)	0.26855 (17)	0.0177 (4)	
C16	0.2942 (2)	0.11820 (13)	0.2545 (2)	0.0297 (4)	
H16	0.2956	0.1555	0.1947	0.036*	
C11	0.13007 (17)	0.26038 (11)	0.24228 (17)	0.0184 (4)	
H11A	0.0888	0.2432	0.1550	0.022*	
H11B	0.2158	0.2780	0.2547	0.022*	
C18	0.03699 (18)	0.63203 (11)	0.17068 (19)	0.0227 (4)	
C19	−0.13340 (17)	0.49154 (11)	0.18207 (19)	0.0220 (4)	
H19A	−0.1551	0.4673	0.2478	0.033*	
H19B	−0.1781	0.4649	0.1032	0.033*	
H19C	−0.1576	0.5467	0.1744	0.033*	
C4	0.05507 (17)	0.54636 (11)	0.13572 (19)	0.0202 (4)	
H4	0.1472	0.5387	0.1585	0.024*	
C15	0.33360 (19)	0.01185 (12)	0.3722 (2)	0.0296 (4)	
H15	0.3667	−0.0370	0.4078	0.036*	
O6	0.07866 (12)	0.32321 (8)	−0.14752 (12)	0.0217 (3)	
O2	−0.04114 (12)	0.43277 (8)	−0.16960 (12)	0.0213 (3)	
O4	0.17443 (12)	0.21504 (8)	0.46368 (12)	0.0229 (3)	
O1	−0.08635 (12)	0.33368 (8)	−0.00508 (12)	0.0223 (3)	
O7	0.11380 (13)	0.66586 (8)	0.25955 (14)	0.0309 (3)	
O8	−0.07111 (13)	0.66571 (8)	0.09307 (13)	0.0281 (3)	
H8A	−0.0768	0.7108	0.1179	0.042*	
O3	0.15754 (12)	0.29677 (8)	0.60630 (12)	0.0246 (3)	
O5	0.36018 (14)	0.04804 (9)	0.27771 (16)	0.0341 (3)	
O4'	0.33614 (12)	0.44634 (7)	0.94502 (12)	0.0201 (3)	
O3'	0.34121 (12)	0.36271 (7)	1.09451 (11)	0.0196 (3)	
O2'	0.58986 (12)	0.23688 (7)	0.49500 (12)	0.0196 (3)	
O1'	0.62849 (12)	0.33263 (8)	0.69732 (12)	0.0220 (3)	
O8'	0.61511 (12)	0.00106 (7)	0.76991 (13)	0.0221 (3)	
H8'	0.6248	−0.0429	0.8036	0.033*	
O6'	0.45406 (13)	0.33889 (8)	0.41371 (13)	0.0287 (3)	
O7'	0.42832 (12)	−0.00338 (7)	0.79601 (14)	0.0253 (3)	
O5'	0.12409 (13)	0.60011 (8)	0.65291 (13)	0.0253 (3)	
C17'	0.35561 (16)	0.37244 (10)	0.99497 (17)	0.0173 (4)	
C9'	0.47726 (16)	0.33266 (10)	0.85748 (16)	0.0166 (4)	
C3'	0.55835 (18)	0.13668 (10)	0.63141 (18)	0.0183 (4)	
H3'1	0.6498	0.1341	0.6789	0.022*	
H3'2	0.5364	0.0977	0.5638	0.022*	
C21'	0.54660 (18)	0.30114 (11)	0.41882 (17)	0.0211 (4)	
C1'	0.55327 (16)	0.27937 (10)	0.68357 (16)	0.0165 (3)	
C13'	0.30056 (17)	0.53252 (10)	0.76765 (17)	0.0184 (4)	
C16'	0.17621 (19)	0.54538 (11)	0.74745 (19)	0.0232 (4)	
H16'	0.1320	0.5205	0.7917	0.028*	
C14'	0.32831 (18)	0.58268 (11)	0.67923 (19)	0.0233 (4)	
H14'	0.4063	0.5874	0.6695	0.028*	
C12'	0.39011 (17)	0.47249 (11)	0.85204 (17)	0.0185 (4)	
H12'	0.4720	0.4988	0.8979	0.022*	
C4'	0.48608 (17)	0.11855 (10)	0.71910 (17)	0.0180 (4)	
H4'	0.3949	0.1247	0.6677	0.022*	
C11'	0.41363 (17)	0.40455 (10)	0.77524 (17)	0.0175 (4)	
H11C	0.4677	0.4233	0.7318	0.021*	
H11D	0.3327	0.3881	0.7114	0.021*	
C5'	0.51693 (16)	0.17822 (10)	0.83185 (17)	0.0170 (4)	
C15'	0.22000 (19)	0.62152 (11)	0.61333 (19)	0.0241 (4)	
H15'	0.2113	0.6581	0.5493	0.029*	
C6'	0.42909 (18)	0.16156 (11)	0.90570 (18)	0.0210 (4)	
H6'1	0.3432	0.1515	0.8453	0.025*	
H6'2	0.4585	0.1136	0.9555	0.025*	
C10'	0.48050 (16)	0.26283 (10)	0.76984 (16)	0.0160 (4)	
H10'	0.3914	0.2571	0.7116	0.019*	
C22'	0.62893 (19)	0.31710 (12)	0.34423 (19)	0.0250 (4)	
H22D	0.5924	0.3596	0.2853	0.037*	
H22E	0.6341	0.2700	0.2987	0.037*	
H22F	0.7131	0.3320	0.4008	0.037*	
C8'	0.38328 (17)	0.30620 (10)	0.91970 (17)	0.0179 (4)	
H8'1	0.3022	0.2948	0.8499	0.021*	
C19'	0.65936 (17)	0.17049 (12)	0.92042 (18)	0.0225 (4)	
H19D	0.6715	0.1930	1.0017	0.034*	
H19E	0.7117	0.1984	0.8841	0.034*	
H19F	0.6831	0.1153	0.9304	0.034*	
C18'	0.50473 (17)	0.03240 (11)	0.76516 (17)	0.0183 (4)	
C7'	0.42364 (19)	0.22877 (11)	0.99402 (18)	0.0223 (4)	
H7'1	0.3630	0.2153	1.0329	0.027*	
H7'2	0.5070	0.2355	1.0609	0.027*	
C20'	0.60724 (17)	0.35762 (11)	0.95727 (18)	0.0213 (4)	
H20D	0.5946	0.3991	1.0095	0.032*	
H20E	0.6615	0.3767	0.9155	0.032*	
H20F	0.6467	0.3126	1.0091	0.032*	

### Atomic displacement parameters (Å^2^)

**Table:**

	*U*^11^	*U*^22^	*U*^33^	*U*^12^	*U*^13^	*U*^23^
C17	0.0127 (8)	0.0244 (9)	0.0235 (10)	0.0000 (7)	0.0056 (7)	−0.0009 (8)
C21	0.0201 (9)	0.0183 (9)	0.0200 (9)	−0.0027 (7)	0.0112 (7)	0.0016 (8)
C10	0.0137 (8)	0.0179 (9)	0.0200 (9)	−0.0004 (7)	0.0066 (7)	−0.0011 (7)
C2	0.0213 (9)	0.0199 (9)	0.0175 (9)	0.0011 (7)	0.0057 (7)	−0.0005 (8)
C13	0.0174 (9)	0.0194 (9)	0.0232 (10)	−0.0038 (7)	0.0046 (7)	−0.0038 (8)
C3	0.0238 (9)	0.0183 (9)	0.0231 (9)	0.0012 (7)	0.0092 (7)	0.0023 (8)
C2'	0.0171 (9)	0.0194 (9)	0.0201 (9)	0.0009 (7)	0.0086 (7)	0.0024 (8)
C7	0.0256 (10)	0.0236 (9)	0.0198 (10)	0.0015 (8)	0.0084 (8)	−0.0037 (8)
C1	0.0154 (9)	0.0159 (9)	0.0252 (10)	0.0032 (7)	0.0092 (7)	−0.0010 (8)
C14	0.0279 (10)	0.0199 (9)	0.0272 (10)	−0.0013 (8)	0.0070 (8)	−0.0010 (8)
C6	0.0223 (9)	0.0205 (9)	0.0222 (9)	−0.0002 (7)	0.0069 (7)	−0.0053 (8)
C20	0.0180 (9)	0.0213 (9)	0.0210 (9)	−0.0006 (7)	0.0074 (7)	0.0021 (8)
C12	0.0188 (9)	0.0201 (9)	0.0197 (9)	−0.0014 (7)	0.0062 (7)	−0.0015 (7)
C5	0.0172 (9)	0.0176 (9)	0.0215 (9)	0.0008 (7)	0.0070 (7)	−0.0022 (8)
C8	0.0170 (9)	0.0217 (9)	0.0193 (9)	−0.0005 (7)	0.0064 (7)	0.0009 (8)
C22	0.0222 (9)	0.0272 (10)	0.0217 (9)	−0.0007 (8)	0.0108 (7)	0.0002 (8)
C9	0.0167 (8)	0.0181 (9)	0.0190 (9)	−0.0011 (7)	0.0076 (7)	−0.0012 (8)
C16	0.0296 (11)	0.0256 (10)	0.0369 (12)	0.0056 (8)	0.0160 (9)	0.0047 (9)
C11	0.0165 (8)	0.0203 (9)	0.0188 (9)	−0.0008 (7)	0.0071 (7)	−0.0023 (7)
C18	0.0200 (10)	0.0205 (9)	0.0285 (11)	−0.0002 (7)	0.0101 (8)	0.0022 (8)
C19	0.0185 (9)	0.0209 (9)	0.0286 (10)	0.0019 (7)	0.0111 (8)	−0.0006 (8)
C4	0.0160 (9)	0.0183 (9)	0.0264 (10)	−0.0006 (7)	0.0082 (7)	−0.0015 (8)
C15	0.0293 (11)	0.0166 (9)	0.0384 (12)	0.0013 (8)	0.0074 (9)	−0.0012 (9)
O6	0.0187 (6)	0.0196 (6)	0.0267 (7)	0.0009 (5)	0.0083 (5)	0.0008 (6)
O2	0.0253 (7)	0.0201 (6)	0.0174 (7)	0.0037 (5)	0.0069 (5)	0.0017 (5)
O4	0.0267 (7)	0.0221 (7)	0.0199 (7)	0.0041 (5)	0.0087 (5)	0.0027 (6)
O1	0.0206 (6)	0.0242 (7)	0.0212 (7)	−0.0027 (5)	0.0069 (5)	−0.0010 (6)
O7	0.0258 (7)	0.0225 (7)	0.0362 (8)	−0.0001 (6)	0.0021 (6)	−0.0070 (7)
O8	0.0260 (7)	0.0161 (6)	0.0354 (8)	0.0042 (5)	0.0039 (6)	−0.0037 (6)
O3	0.0232 (7)	0.0309 (7)	0.0199 (7)	0.0024 (5)	0.0084 (5)	0.0012 (6)
O5	0.0321 (8)	0.0276 (8)	0.0475 (9)	0.0056 (6)	0.0203 (7)	−0.0017 (7)
O4'	0.0247 (7)	0.0172 (6)	0.0204 (7)	0.0027 (5)	0.0107 (5)	0.0005 (5)
O3'	0.0228 (7)	0.0198 (6)	0.0177 (6)	−0.0008 (5)	0.0092 (5)	−0.0016 (5)
O2'	0.0233 (7)	0.0178 (6)	0.0203 (7)	0.0031 (5)	0.0112 (5)	0.0027 (5)
O1'	0.0227 (6)	0.0178 (6)	0.0292 (7)	−0.0028 (5)	0.0139 (6)	−0.0011 (6)
O8'	0.0239 (7)	0.0146 (6)	0.0318 (7)	0.0032 (5)	0.0149 (6)	0.0057 (6)
O6'	0.0266 (7)	0.0284 (7)	0.0334 (8)	0.0101 (6)	0.0138 (6)	0.0108 (6)
O7'	0.0249 (7)	0.0172 (6)	0.0395 (8)	−0.0016 (5)	0.0184 (6)	0.0005 (6)
O5'	0.0257 (7)	0.0217 (7)	0.0292 (7)	0.0066 (5)	0.0111 (6)	0.0031 (6)
C17'	0.0132 (8)	0.0174 (9)	0.0187 (9)	−0.0002 (6)	0.0030 (7)	−0.0002 (8)
C9'	0.0165 (8)	0.0155 (8)	0.0183 (9)	−0.0014 (7)	0.0071 (7)	−0.0020 (7)
C3'	0.0212 (9)	0.0148 (8)	0.0200 (9)	0.0003 (7)	0.0088 (7)	−0.0021 (7)
C21'	0.0239 (9)	0.0187 (9)	0.0183 (9)	0.0005 (7)	0.0051 (7)	0.0000 (7)
C1'	0.0151 (8)	0.0146 (8)	0.0190 (9)	0.0037 (7)	0.0055 (7)	0.0043 (7)
C13'	0.0243 (10)	0.0122 (8)	0.0193 (9)	−0.0006 (7)	0.0088 (7)	−0.0045 (7)
C16'	0.0292 (10)	0.0191 (9)	0.0250 (10)	0.0042 (8)	0.0145 (8)	0.0033 (8)
C14'	0.0247 (10)	0.0189 (9)	0.0287 (11)	−0.0008 (8)	0.0126 (8)	0.0007 (8)
C12'	0.0179 (9)	0.0170 (9)	0.0207 (9)	−0.0030 (7)	0.0075 (7)	−0.0018 (7)
C4'	0.0158 (9)	0.0162 (9)	0.0225 (9)	−0.0004 (7)	0.0079 (7)	0.0010 (8)
C11'	0.0192 (9)	0.0163 (8)	0.0193 (9)	−0.0012 (7)	0.0096 (7)	−0.0006 (7)
C5'	0.0180 (9)	0.0141 (8)	0.0187 (9)	0.0006 (7)	0.0069 (7)	0.0005 (7)
C15'	0.0329 (11)	0.0156 (9)	0.0261 (10)	−0.0005 (8)	0.0138 (8)	0.0015 (8)
C6'	0.0273 (10)	0.0149 (8)	0.0230 (9)	0.0016 (7)	0.0121 (8)	0.0030 (8)
C10'	0.0135 (8)	0.0154 (9)	0.0180 (9)	0.0003 (6)	0.0045 (7)	0.0007 (7)
C22'	0.0278 (10)	0.0249 (10)	0.0232 (10)	0.0016 (8)	0.0108 (8)	0.0059 (8)
C8'	0.0172 (8)	0.0178 (9)	0.0194 (9)	−0.0003 (7)	0.0077 (7)	−0.0008 (7)
C19'	0.0233 (10)	0.0189 (9)	0.0219 (10)	0.0037 (7)	0.0045 (7)	−0.0004 (8)
C18'	0.0212 (9)	0.0154 (9)	0.0187 (9)	−0.0017 (7)	0.0079 (7)	−0.0035 (7)
C7'	0.0275 (10)	0.0196 (9)	0.0227 (10)	0.0035 (7)	0.0128 (8)	0.0017 (8)
C20'	0.0198 (9)	0.0190 (9)	0.0242 (10)	−0.0003 (7)	0.0070 (7)	−0.0041 (8)

### Geometric parameters (Å, °)

**Table:**

C17—O3	1.208 (2)	C4—H4	0.980
C17—O4	1.349 (2)	C15—O5	1.362 (3)
C17—C8	1.512 (3)	C15—H15	0.930
C21—O6	1.213 (2)	O8—H8A	0.820
C21—O2	1.348 (2)	O4'—C17'	1.349 (2)
C21—C22	1.488 (3)	O4'—C12'	1.471 (2)
C10—C1	1.504 (3)	O3'—C17'	1.214 (2)
C10—C9	1.546 (3)	O2'—C21'	1.356 (2)
C10—C5	1.582 (2)	O1'—C1'	1.202 (2)
C10—H10	0.980	O8'—C18'	1.334 (2)
C2—O2	1.440 (2)	O8'—H8'	0.820
C2—C1	1.523 (3)	O6'—C21'	1.204 (2)
C2—C3	1.525 (3)	O7'—C18'	1.205 (2)
C2—H2	0.980	O5'—C15'	1.366 (2)
C13—C16	1.345 (3)	O5'—C16'	1.370 (2)
C13—C14	1.431 (3)	C17'—C8'	1.506 (2)
C13—C12	1.495 (3)	C9'—C11'	1.532 (2)
C3—C4	1.536 (3)	C9'—C20'	1.539 (2)
C3—H3A	0.970	C9'—C8'	1.543 (2)
C3—H3B	0.970	C9'—C10'	1.549 (2)
C2'—O2'	1.434 (2)	C3'—C4'	1.536 (2)
C2'—C1'	1.517 (3)	C3'—H3'1	0.970
C2'—C3'	1.526 (2)	C3'—H3'2	0.970
C2'—H2'	0.980	C21'—C22'	1.497 (3)
C7—C6	1.527 (3)	C1'—C10'	1.522 (2)
C7—C8	1.527 (3)	C13'—C16'	1.351 (3)
C7—H7A	0.970	C13'—C14'	1.434 (3)
C7—H7B	0.970	C13'—C12'	1.495 (3)
C1—O1	1.206 (2)	C16'—H16'	0.930
C14—C15	1.337 (3)	C14'—C15'	1.340 (3)
C14—H14	0.930	C14'—H14'	0.930
C6—C5	1.539 (2)	C12'—C11'	1.520 (2)
C6—H6A	0.970	C12'—H12'	0.980
C6—H6B	0.970	C4'—C18'	1.527 (2)
C20—C9	1.543 (2)	C4'—C5'	1.561 (3)
C20—H20A	0.960	C4'—H4'	0.980
C20—H20B	0.960	C11'—H11C	0.970
C20—H20C	0.960	C11'—H11D	0.970
C12—O4	1.471 (2)	C5'—C6'	1.545 (2)
C12—C11	1.513 (3)	C5'—C19'	1.550 (2)
C12—H12	0.980	C5'—C10'	1.572 (2)
C5—C19	1.544 (2)	C15'—H15'	0.930
C5—C4	1.567 (3)	C6'—C7'	1.528 (3)
C8—C9	1.541 (2)	C6'—H6'1	0.970
C8—H8	0.980	C6'—H6'2	0.970
C22—H22A	0.960	C10'—H10'	0.980
C22—H22B	0.960	C22'—H22D	0.960
C22—H22C	0.960	C22'—H22E	0.960
C9—C11	1.546 (2)	C22'—H22F	0.960
C16—O5	1.366 (3)	C8'—C7'	1.525 (2)
C16—H16	0.930	C8'—H8'1	0.980
C11—H11A	0.970	C19'—H19D	0.960
C11—H11B	0.970	C19'—H19E	0.960
C18—O7	1.200 (2)	C19'—H19F	0.960
C18—O8	1.335 (2)	C7'—H7'1	0.970
C18—C4	1.527 (3)	C7'—H7'2	0.970
C19—H19A	0.960	C20'—H20D	0.960
C19—H19B	0.960	C20'—H20E	0.960
C19—H19C	0.960	C20'—H20F	0.960
			
O3—C17—O4	117.51 (17)	C14—C15—H15	124.6
O3—C17—C8	123.97 (17)	O5—C15—H15	124.6
O4—C17—C8	118.25 (15)	C21—O2—C2	114.88 (14)
O6—C21—O2	121.98 (16)	C17—O4—C12	122.60 (14)
O6—C21—C22	126.11 (17)	C18—O8—H8A	109.5
O2—C21—C22	111.91 (16)	C15—O5—C16	105.86 (16)
C1—C10—C9	115.10 (15)	C17'—O4'—C12'	122.14 (14)
C1—C10—C5	108.90 (14)	C21'—O2'—C2'	114.68 (14)
C9—C10—C5	117.10 (15)	C18'—O8'—H8'	109.5
C1—C10—H10	104.8	C15'—O5'—C16'	106.00 (14)
C9—C10—H10	104.8	O3'—C17'—O4'	117.37 (16)
C5—C10—H10	104.8	O3'—C17'—C8'	124.00 (16)
O2—C2—C1	110.60 (14)	O4'—C17'—C8'	118.44 (15)
O2—C2—C3	107.88 (15)	C11'—C9'—C20'	109.58 (14)
C1—C2—C3	110.20 (15)	C11'—C9'—C8'	105.19 (14)
O2—C2—H2	109.4	C20'—C9'—C8'	111.58 (14)
C1—C2—H2	109.4	C11'—C9'—C10'	108.80 (14)
C3—C2—H2	109.4	C20'—C9'—C10'	115.87 (14)
C16—C13—C14	105.66 (17)	C8'—C9'—C10'	105.21 (14)
C16—C13—C12	128.21 (18)	C2'—C3'—C4'	108.73 (14)
C14—C13—C12	126.05 (17)	C2'—C3'—H3'1	109.9
C2—C3—C4	109.27 (15)	C4'—C3'—H3'1	109.9
C2—C3—H3A	109.8	C2'—C3'—H3'2	109.9
C4—C3—H3A	109.8	C4'—C3'—H3'2	109.9
C2—C3—H3B	109.8	H3'1—C3'—H3'2	108.3
C4—C3—H3B	109.8	O6'—C21'—O2'	123.28 (17)
H3A—C3—H3B	108.3	O6'—C21'—C22'	126.00 (17)
O2'—C2'—C1'	111.40 (14)	O2'—C21'—C22'	110.72 (16)
O2'—C2'—C3'	108.58 (14)	O1'—C1'—C2'	122.59 (16)
C1'—C2'—C3'	108.88 (15)	O1'—C1'—C10'	125.18 (16)
O2'—C2'—H2'	109.3	C2'—C1'—C10'	112.22 (14)
C1'—C2'—H2'	109.3	C16'—C13'—C14'	105.75 (16)
C3'—C2'—H2'	109.3	C16'—C13'—C12'	128.77 (17)
C6—C7—C8	109.98 (15)	C14'—C13'—C12'	125.13 (16)
C6—C7—H7A	109.7	C13'—C16'—O5'	110.76 (16)
C8—C7—H7A	109.7	C13'—C16'—H16'	124.6
C6—C7—H7B	109.7	O5'—C16'—H16'	124.6
C8—C7—H7B	109.7	C15'—C14'—C13'	106.67 (17)
H7A—C7—H7B	108.2	C15'—C14'—H14'	126.7
O1—C1—C10	125.78 (17)	C13'—C14'—H14'	126.7
O1—C1—C2	122.15 (17)	O4'—C12'—C13'	107.57 (14)
C10—C1—C2	112.04 (15)	O4'—C12'—C11'	113.40 (14)
C15—C14—C13	106.63 (18)	C13'—C12'—C11'	111.01 (15)
C15—C14—H14	126.7	O4'—C12'—H12'	108.2
C13—C14—H14	126.7	C13'—C12'—H12'	108.2
C7—C6—C5	113.95 (15)	C11'—C12'—H12'	108.2
C7—C6—H6A	108.8	C18'—C4'—C3'	111.86 (14)
C5—C6—H6A	108.8	C18'—C4'—C5'	111.53 (15)
C7—C6—H6B	108.8	C3'—C4'—C5'	113.38 (14)
C5—C6—H6B	108.8	C18'—C4'—H4'	106.5
H6A—C6—H6B	107.7	C3'—C4'—H4'	106.5
C9—C20—H20A	109.5	C5'—C4'—H4'	106.5
C9—C20—H20B	109.5	C12'—C11'—C9'	112.55 (14)
H20A—C20—H20B	109.5	C12'—C11'—H11C	109.1
C9—C20—H20C	109.5	C9'—C11'—H11C	109.1
H20A—C20—H20C	109.5	C12'—C11'—H11D	109.1
H20B—C20—H20C	109.5	C9'—C11'—H11D	109.1
O4—C12—C13	105.53 (14)	H11C—C11'—H11D	107.8
O4—C12—C11	113.56 (14)	C6'—C5'—C19'	110.49 (15)
C13—C12—C11	115.33 (15)	C6'—C5'—C4'	109.82 (14)
O4—C12—H12	107.3	C19'—C5'—C4'	109.82 (14)
C13—C12—H12	107.3	C6'—C5'—C10'	107.14 (14)
C11—C12—H12	107.3	C19'—C5'—C10'	113.65 (14)
C6—C5—C19	110.08 (14)	C4'—C5'—C10'	105.75 (14)
C6—C5—C4	109.75 (15)	C14'—C15'—O5'	110.82 (17)
C19—C5—C4	109.75 (15)	C14'—C15'—H15'	124.6
C6—C5—C10	108.25 (14)	O5'—C15'—H15'	124.6
C19—C5—C10	113.76 (14)	C7'—C6'—C5'	114.53 (15)
C4—C5—C10	105.10 (14)	C7'—C6'—H6'1	108.6
C17—C8—C7	113.59 (15)	C5'—C6'—H6'1	108.6
C17—C8—C9	112.03 (15)	C7'—C6'—H6'2	108.6
C7—C8—C9	112.36 (15)	C5'—C6'—H6'2	108.6
C17—C8—H8	106.1	H6'1—C6'—H6'2	107.6
C7—C8—H8	106.1	C1'—C10'—C9'	115.14 (14)
C9—C8—H8	106.1	C1'—C10'—C5'	109.95 (14)
C21—C22—H22A	109.5	C9'—C10'—C5'	117.47 (14)
C21—C22—H22B	109.5	C1'—C10'—H10'	104.2
H22A—C22—H22B	109.5	C9'—C10'—H10'	104.2
C21—C22—H22C	109.5	C5'—C10'—H10'	104.2
H22A—C22—H22C	109.5	C21'—C22'—H22D	109.5
H22B—C22—H22C	109.5	C21'—C22'—H22E	109.5
C8—C9—C20	111.05 (14)	H22D—C22'—H22E	109.5
C8—C9—C10	106.71 (14)	C21'—C22'—H22F	109.5
C20—C9—C10	114.89 (14)	H22D—C22'—H22F	109.5
C8—C9—C11	105.14 (14)	H22E—C22'—H22F	109.5
C20—C9—C11	109.83 (15)	C17'—C8'—C7'	113.41 (14)
C10—C9—C11	108.72 (14)	C17'—C8'—C9'	111.80 (14)
C13—C16—O5	110.99 (19)	C7'—C8'—C9'	112.71 (15)
C13—C16—H16	124.5	C17'—C8'—H8'1	106.1
O5—C16—H16	124.5	C7'—C8'—H8'1	106.1
C12—C11—C9	110.38 (15)	C9'—C8'—H8'1	106.1
C12—C11—H11A	109.6	C5'—C19'—H19D	109.5
C9—C11—H11A	109.6	C5'—C19'—H19E	109.5
C12—C11—H11B	109.6	H19D—C19'—H19E	109.5
C9—C11—H11B	109.6	C5'—C19'—H19F	109.5
H11A—C11—H11B	108.1	H19D—C19'—H19F	109.5
O7—C18—O8	123.35 (18)	H19E—C19'—H19F	109.5
O7—C18—C4	122.76 (17)	O7'—C18'—O8'	123.29 (17)
O8—C18—C4	113.89 (16)	O7'—C18'—C4'	123.43 (16)
C5—C19—H19A	109.5	O8'—C18'—C4'	113.26 (15)
C5—C19—H19B	109.5	C8'—C7'—C6'	110.04 (15)
H19A—C19—H19B	109.5	C8'—C7'—H7'1	109.7
C5—C19—H19C	109.5	C6'—C7'—H7'1	109.7
H19A—C19—H19C	109.5	C8'—C7'—H7'2	109.7
H19B—C19—H19C	109.5	C6'—C7'—H7'2	109.7
C18—C4—C3	112.41 (16)	H7'1—C7'—H7'2	108.2
C18—C4—C5	111.84 (15)	C9'—C20'—H20D	109.5
C3—C4—C5	112.27 (15)	C9'—C20'—H20E	109.5
C18—C4—H4	106.6	H20D—C20'—H20E	109.5
C3—C4—H4	106.6	C9'—C20'—H20F	109.5
C5—C4—H4	106.6	H20D—C20'—H20F	109.5
C14—C15—O5	110.86 (18)	H20E—C20'—H20F	109.5

### Hydrogen-bond geometry (Å, °)

**Table:**

*D*—H···*A*	*D*—H	H···*A*	*D*···*A*	*D*—H···*A*
O8—H8A···O6^i^	0.82	1.92	2.7264 (18)	168
O8'—H8'···O3'^ii^	0.82	1.92	2.7308 (17)	172

Symmetry codes: (i) −*x*, *y*+1/2, −*z*; (ii) −*x*+1, *y*−1/2, −*z*+2.
